# EPIDEMIOLOGIC PATTERNS OF POTENTIALLY INAPPROPRIATE MEDICATION USE IN OLDER ADULT DRIVERS

**DOI:** 10.1093/geroni/igad104.3305

**Published:** 2023-12-21

**Authors:** Jesse Cotton, Beicheng Huang, Stanford Chihuri, Barbara Lang, Guohua Li

**Affiliations:** Columbia University Vagelos College of Physicians & Surgeons, New York City, New York, United States; Columbia University, New York City, New York, United States; Columbia University, New York City, New York, United States; Columbia University, New York City, New York, United States; Columbia University, New York City, New York, United States

## Abstract

Use of potentially inappropriate medications (PIMs) by older adult drivers has been linked to increased crash risk. The objective of this study was to examine the epidemiologic patterns of PIM use in a cohort of 2990 drivers aged 65-79 years at the time enrollment (July 2015 to March 2017). We applied the 2023 Beers Criteria to identify PIMs from medication data collected through the “brown-bag review” method at baseline, year 2 and year 4 of follow-up for older adults participating in the Longitudinal Research on Aging Drivers project. We assessed time trends in the prevalence of PIM use and the composition of PIMs using the chi-square tests. The prevalence of PIM use was 16.2% at baseline, which decreased slightly in year 2 (14.0%) and year 4 (13.2%). The three most frequently used PIMs were nonbenzodiazepine hypnotics, antidepressants, and estrogens, accounting for 19.4%, 17.8% and 14.1% of all PIMs, respectively. Of PIM users, 12.7% were using ≥2 PIMs at baseline, 12.5% in year 2, and 10.0% in year 4. PIM use among older adult drivers is fairly common. Epidemiologic patterns of PIM use among older adult drivers have remained stable during the four years of follow-up.

